# Antiviral Properties against SARS-CoV-2 of Nanostructured ZnO Obtained by Green Combustion Synthesis and Coated in Waterborne Acrylic Coatings

**DOI:** 10.3390/nano12234345

**Published:** 2022-12-06

**Authors:** Julia de O. Primo, Jamille de S. Correa, Dienifer F. L. Horsth, Arkaprava Das, Marcin Zając, Polona Umek, Ruddy Wattiez, Fauze J. Anaissi, Rob C. A. Onderwater, Carla Bittencourt

**Affiliations:** 1 Departamento de Química, Universidade Estadual Do Centro-Oeste, Guarapuava 85-040-200, Brazil; 2 Chimie des Interactions Plasma-Surface (ChIPS), Research Institute for Materials Science and Engineering, University of Mons, 7000 Mons, Belgium; 3 National Synchrotron Radiation Centre Solaris, Jagiellonian University, 30-392 Kraków, Poland; 4 Solid State Physics Department, Jožef Stefan Institute, 1000 Ljubljana, Slovenia; 5 Department of Proteomics and Microbiology, University of Mons, 7000 Mons, Belgium; 6 Materia Nova ASBL, 7000 Mons, Belgium

**Keywords:** zinc oxide, eco-friendly synthesis, coating, SARS-CoV-2, coronavirus, antiviral surface

## Abstract

The COVID-19 pandemic has increased the need for developing disinfectant surfaces as well as reducing the spread of infections on contaminated surfaces and the contamination risk from the fomite route. The present work reports on the antiviral activity of coatings containing ZnO particles obtained by two simple synthesis routes using *Aloe vera* (ZnO-aloe) or cassava starch (ZnO-starch) as reaction fuel. After detailed characterization using XRD and NEXAFS, the obtained ZnO particles were dispersed in a proportion of 10% with two different waterborne acrylic coatings (binder and commercial white paint) and brushed on the surface of polycarbonates (PC). The cured ZnO/coatings were characterized by scanning electron microscopes (SEM) and energy-dispersive X-ray spectroscopy (EDS). Wettability tests were performed. The virucidal activity of the ZnO particles dispersed in the waterborne acrylic coating was compared to a reference control sample (PC plates). According to RT-PCR results, the ZnO-aloe/coating displays the highest outcome for antiviral activity against SARS-CoV-2 using the acrylic binder, inactivating >99% of the virus after 24 h of contact relative to reference control.

## 1. Introduction

Since 2019, the coronavirus disease (COVID-19) has been a burden on healthcare systems and economies around the Globe, with more than 590 million reported cases and nearly 6 million deaths [1]. The severe accurate respiratory syndrome coronavirus 2 (SARS-CoV-2) is a single-stranded positive-strand RNA virus with an envelope and belongs to the family of beta coronaviruses [2]. These respiratory RNA viruses are highly contagious, and the primary transmission mode is through exposure to respiratory droplets [3]. Another route of SARS-CoV-2 transmission may also be the deposition of droplets on surfaces, forming fomites, subsequently assimilated by a person via touch [4]. However, the infectivity of a given droplet-nucleus/fomite is related to the initial viral load and its stability in different environments [5]. Previous work [6] reported that SARS-CoV-2 is viable for up to 72 h on plastic, 48 h on stainless steel, and 4 h on copper foil.

Since touching joint surfaces, i.e., door handles, toilets, taps, and work surfaces, is considered a potential transmission route, the World Health Organization (WHO) recommends surface disinfection to reduce the risk of contamination from the fomite route [7]. This is the most recommended method to reduce the risk of transmission of viruses, including COVID-19 [3]. Chemical-based surface disinfectants such as hydrogen peroxide, sodium hypochlorite, and alcohol are used worldwide for disinfection [8]; however, after disinfection using these products, the surface can be contaminated again by an infected person. As a result, methods for ensuring continuous antiviral protection of surfaces are needed to eliminate viruses shortly after contamination [3]. The study of nanostructured semiconductor-based composites aims to increase biological applications [9,10,11,12]. In this context, an alternative approach is to use nanostructured semiconductor-based coatings designed to provide a self-disinfection, inactivating or killing microbes long after the coating is applied [13]; CuO [5,7], TiO_2_ [2,7,14], and ZnO [7,13] have been reported to inactivate SARS-CoV-2. The virucidal activity using acrylic paint containing galvanic microcells has also been reported [3]. The present study evaluates the virucidal activity of acrylic paint surfaces painted with nanostructured ZnO pigments. Zinc oxide has been reported as antibacterial material, being active against Gram-positive and Gram-negative bacteria [15,16,17], as well as an antiviral material [13,18], which has potential application in different systems such as food packaging [19] and antipathogenic surfaces [20]. ZnO composites have been incorporated into polymers and tested as antiviral/antibacterial surfaces [7,13,20]. Antiviral mechanisms associated with ZnO include preventing viral entry, replication, and spreading to organs, leading to oxidative injury and viral death via reactive oxygen species. The zinc-containing compounds displayed antiviral activity through physical processes, including attachment to the virus, the inhibition of virus infection, and uncoating the virus. These compounds also inhibit viral polymerases and proteases via biological mechanisms [21]. Herein, ZnO particles obtained by eco-friendly routes and applied in an acrylic waterborne coating against SAR-CoV-2 are reported. Firstly, two eco-friendly routes were utilized to obtain the ZnO particles, using two different polysaccharides extracted from *Aloe vera* and cassava starch as high combustion power to reduce the calcination temperature. Secondly, ZnO particles were blended with an acrylic binder and a commercial white paint. Finally, the mixtures painted on polycarbonate surfaces were evaluated for their antiviral properties against SARS-CoV-2 in a control sample, i.e., a polycarbonate surface with no coating.

## 2. Materials and Methods

### 2.1. Materials

Zinc nitrate hexahydrate (Zn(NO_3_)_2_·6H_2_O, 98%, NEON, Suzano, Brazil) was used. All solutions were prepared with deionized water. Natural cassava starch from colloidal suspension and *Aloe vera* extract were used as fuels [22,23]. The process of obtaining the *Aloe vera* extract was reported elsewhere [24].

### 2.2. Synthesis of ZnO Using Polysaccharides

**Figure 1:**
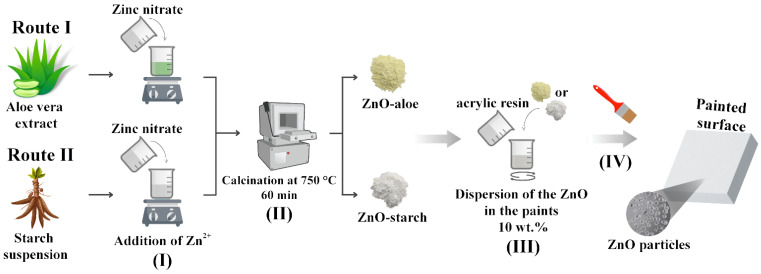
Schema of the different synthetic routes of obtaining nanostructured ZnO and its dispersion in waterborne acrylic coatings. Steps: (I) the addition of the zinc ions to the polysaccharides suspensions; (II) Calcination; (III) dispersion of the pigments in paint; and (IV) the exposure of the surface against the SARS-CoV-2 virus.

The ZnO particles were synthesized using polysaccharides extracted from cassava starch and *Aloe vera* as fuels for the reaction, as described elsewhere [25] (Figure 1). The samples were labeled regarding the fuel utilized, ZnO-aloe and ZnO-starch.

### 2.3. Waterborne Acrylic Coatings

A colorless commercial waterborne acrylic binder with no solids content (Acrylic Binder Amsterdam 005, prod. Royal Talens, Apeldoorn, The Netherlands), and white waterborne paint, with a solid content by weight of 50.5–52.5%, a VOC (volatile organic compounds) < 30 g/dm^3^, and a pH of 8–9 (Paracem^®^ deco matt, prod. Martin Mathys N. V., Zelem, Belgium) were used for the dispersion of ZnO. The coatings were labeled in this work as binder-A and paint-W, respectively. In a proportion of 10 wt.%, ZnO particles were dispersed in the coatings (binder-A and paint-W) and mixed to obtain a uniform dispersion for 30 min in a magnetic stirring. Next, the well-dispersed mixtures were painted on polycarbonate surfaces (PC) using a brush (Figure 1).

### 2.4. Characterization Techniques

The X-ray diffraction (XRD) performed on a D2 Phaser (Bruker, Karlsruhe, Germany) with Cu Kα radiation (λ = 1.5418 Å) was used to identify the crystalline structure of the synthesized ZnO. In addition, the local structure (or the local bonding environment) of the ZnO particles synthesized using the two different routes was investigated by near-edge X-ray absorption fine structure (NEXAFS) recorded at the PEEM/XAS beamline (photon energy range of 200–2000 eV) of the SOLARIS Synchrotron (Krakow—Poland) [26]. NEXAFS measurements were carried out in partial fluorescence yield (PFY) at room temperature for the samples in powder form, and the obtained spectra were normalized using PyMCA software, version 5.7.5 [27].

The morphology of the particulate samples and the ZnO coatings were examined with a field emission scanning electron microscope (FE-SEM, Verios G4, Thermo Fisher, Waltham, MA, USA). For the SEM analysis, the particulate samples were dispersed in water, and a drop of dispersion was deposited on a polished Al sample holder; for investigations of the ZnO coatings, coated silicon plates were mounted to Al sample holders using silver paint. Before the SEM investigation, a ~5-nm-thick carbon layer was deposited on the specimens’ surfaces.

The wettability properties of the surfaces were evaluated by contact angle (CQ) using an optical tensiometer (Attension Theta, Biolin Scientific, Gothenburg, Sweden) and a water droplet with a volume of ~5 µL under ambient conditions (25 °C). In addition, different regions of the surface were analyzed to verify the uniformity.

### 2.5. SARS-CoV-2 Inactivation Test

The inactivation of the SAR-CoV2 virus was measured using the RT-PCR protocol of the national COVID-19 detection service in a procedure for viral inactivation detection described previously [28,29,30]. SARS-CoV2 viral particles were isolated into Hank’s balanced salt solution from nasopharyngeal swabs of confirmed COVID-19 patients and stored at -80°C until application. The samples were residuals from the initial COVID-19 testing platform of UMONS (University of Mons, Belgium) and came to the testing platform from all over the Hainault region in Belgium. To evaluate the antiviral properties of the ZnO-based coatings, PC plates (25 × 25 mm) were coated with the water-acrylic coatings (control samples), and the exposure phase of the surfaces containing the ZnO synthesized against SARS-CoV-2 was done according to the adapted ISO 22196:2011 [31]. The samples were placed in individual discs in quadruplicate.

The coated PC samples were placed in sterile Petri dishes. A liquid volume of 100 μL in a virus concentration corresponding to a Ct value in RT-qPCR of approximately 22 was added to each surface, and a polyethylene film cover of 20 × 20 mm was placed on top of the liquid. The inoculated specimens were then incubated at room temperature for 24 h under humid conditions. After the incubation, intact viral particles were recovered in 200 μL of a viral recovery solution containing 5 M guanidinium thiocyanate, 40 mM dithiothreitol, 20 µg/mL glycogen, and 1% Triton X-100, buffered with 25 mM sodium citrate to pH 8 [32]. The viral RNA was extracted using the manufacturer’s extraction protocol with AMPure XP magnetic beads (Beckman Coulter, Villepinte, France).

SARS-CoV-2 viral suspensions were tested using the RT-PCR kit (ViroReal Kit, Ingenetix GmbH, Wien, Austria). As viral load indicators, the Ct values and the number of cycles necessary to spot the virus were generated via the RT-PCR test. The amplification reactions were performed using TaqMan RT-PCR on a StepOne Plus real-time PCR system (Applied Biosystems, Thermo Fisher, Waltham, MA, USA). The primers used were SARS E_Sarbeco-P1 (FAM-ACACTAGCCATCCTTACTGCGCTTCGBBQ), SARS E_Sarbeco-F1 (ACAGGTACGTTAATAGTTAATAGCGT), and SARS E_Sarbeco-R2 (ATATTGCAGCAGTACGCACACA) for the E gene using the Eurogentec (Belgium) Mastermix containing ROX as the internal reference. The PCR conditions were as follows: the initial denaturation step, 48 °C for 10 min for reverse transcription, followed by 95 °C for 3 min, and then 45 cycles of 95 °C for 15 s, 58 °C for 30 s. Applied Biosystems ViiA7 instruments (Applied Biosystems, Hong Kong, China) were used.

## 3. Results and Discussion

### 3.1. Characterization of ZnO Powder

**Figure 2:**
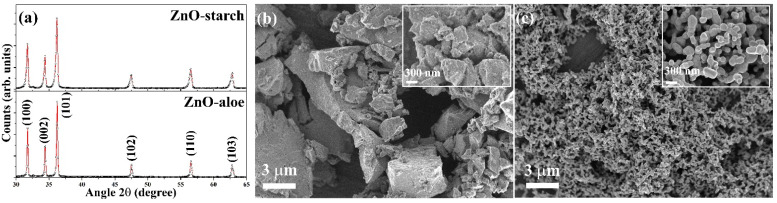
Characterization images of as-prepared ZnO-aloe and ZnO-starch powders: (**a**) X-ray diffraction pattern (showing the hexagonal wurtzite crystal structure of ZnO); (**b**) SEM images of ZnO-aloe at low magnification (inset: SEM images at high magnification); (**c**) SEM images of ZnO-starch at low magnification (inset: SEM images at high magnification).

First, the crystallographic structure and morphology of the synthesized ZnO particles were investigated. The XRD patterns of the ZnO-aloe and ZnO-starch powder samples, shown in Figure 2, follow the ICDD card 01-075-9742 of the hexagonal phase with the Wurtzite structure [25]. The high peak intensities indicate the formation of crystalline ZnO, irrespective of the synthetic route used. The shape and size of the particles in the ZnO-starch and ZnO-aloe powder samples were examined by SEM (Figure 2b,c). SEM revealed that using different fuels (starch and aloe vera) in the synthesis significantly impacted the shape and size of formed particles. In the SEM images of ZnO-starch, particle size ranges from 100 to 350 nm, while ZnO-aloe particles are massive (up to a few μm), with undefined morphology.

**Figure 3:**
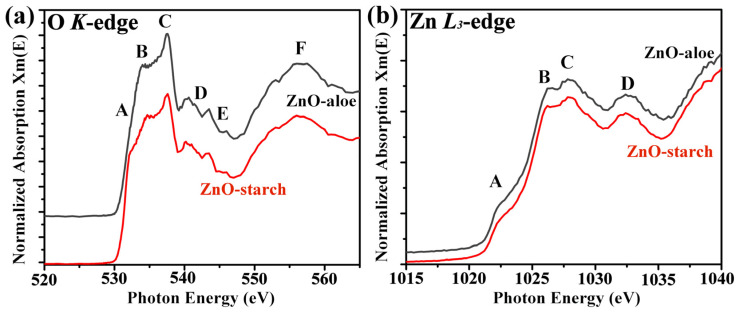
Normalized spectra of (**a**) O *K*-edge and (**b**) Zn *L*_3_-edge NEXAFS spectra of ZnO-aloe and ZnO-starch samples.

Materials obtained using green synthetic routes like the ones in this work are prone to impurities from the plants, which may affect the local electronic structure of the ZnO. To better understand the ZnO particles’ local electronic structure and investigate the presence of defects, NEXAFS analysis of the ZnO-aloe and ZnO-starch powder samples were performed. The normalized O *K*-edge and Zn *L*-edge spectra of ZnO are shown in Figure 3. The O *K*-edge spectra of both ZnO powder samples show broad, asymmetric spectral features denoted by A, B, C, D, E, and F. The overall spectral features in ~530–539 eV were assigned to the transition of O 1s electrons to the hybridized orbitals of O 2p and Zn 4s states [33,34]. The region of ~539–550 eV (labeled by D–E) is associated with transitions to the states formed by the hybridization between the O 2p states with the Zn 4p states [33], and above 550 eV (indicated by F); the contribution mainly comes from O 2p–Zn 4d [35]. The sharp peak (indicated by C) near 537 eV is due to the transition of O 1s electrons to more localized O 2pz and 2px + y states [33,34].

There are some systematic changes in the features of the O *K*-edge spectra depending on the synthetic route used: for the ZnO-starch sample, a feature can be observed at 532.3 eV (indicated by A). This pre-edge feature was associated with O vacancies and other defects [33,34,36]. Therefore, ZnO-starch has higher O vacancies, which may be introduced during the combustion synthesis.

The Zn *L*_3_-edge spectra recorded on the ZnO-aloe and ZnO-starch powder samples are shown in Figure 3b. The Zn *L*_3_-edge is composed of four peaks at 1022.4, 1026.3, 1028, and 1032.4 eV, which are assigned as A, B, C, and D; according to the dipole-transition selection rules, the Zn *L*_3_-edge XAS spectra reflect the unoccupied Zn *s*- and *d*-derived states [37,38]. Since Zn 3d is occupied, Zn 4s is the lowest unoccupied orbital, followed by Zn 4p and 4d [37]. The pre-edge peak at 1022.4 corresponds to the Zn–4 *s* derived state [34]. In contrast, the other peaks (B and C) are mainly related to the transition of 2p electrons to the Zn 4p levels [39]. These results confirm that both synthetic routes led to the synthesis of crystalline ZnO once *L*-edge gives the fingerprint of the ZnO crystallographic phase. Furthermore, both NEXAFS spectra are similar to those reported in the literature for ZnO hexagonal phase with Wurtzite structure, in agreement with the XRD analysis [38,40].

### 3.2. Surface Characterization of ZnO Coatings

According to the United States Food and Drug Administration (FDA), zinc oxide (ZnO) is classified as “Generally Regarded as Safe” (GRAS) for various biological applications [41,42]; nevertheless, further toxicological studies considering aspects such as particle morphology, size, and concentration are necessary to test the biocompatibility of ZnO-based nanomaterials for biomedical applications such as drug delivery antibacterial materials, wound healing, tissue engineering, etc. [43,44,45,46,47,48]. In addition to antipathogenic properties when used as pigments, nanostructured ZnO does not cause color variations when added to paints and coatings [49]. In addition, when embedded as particles within polymer matrices, such as polyethylene glycol, its cytotoxicity was reported to decrease, as the release of Zn^2+^ ions and reactive oxygen species are prevented without reducing the antipathogenic properties [18]. In the case of this present research, the zinc oxide particles were enforced in an acrylic-based binder, and white acrylic paint was evaluated as a virucidal coating. The synthesized ZnO powders were mixed with the commercial paints (binder-A and paint-W) in 10 wt.% and brushed on polycarbonate (PC) plates.

**Figure 4:**
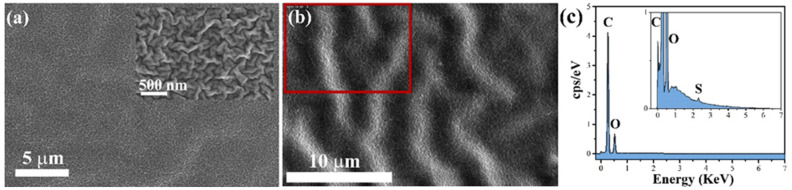
SEM images of acrylic binder surface (binder-A) non-loaded ZnO particles at (**a**) low magnification (inset: SEM images at high magnification); (**b**) BSE-SEM image of binder-A; (**c**) EDS spectra from the binder-A surface. The inset shows the EDS spectra focused on the Sulfur region. The red frame in the BSE-SEM image indicates the area of analysis.

**Figure 5:**
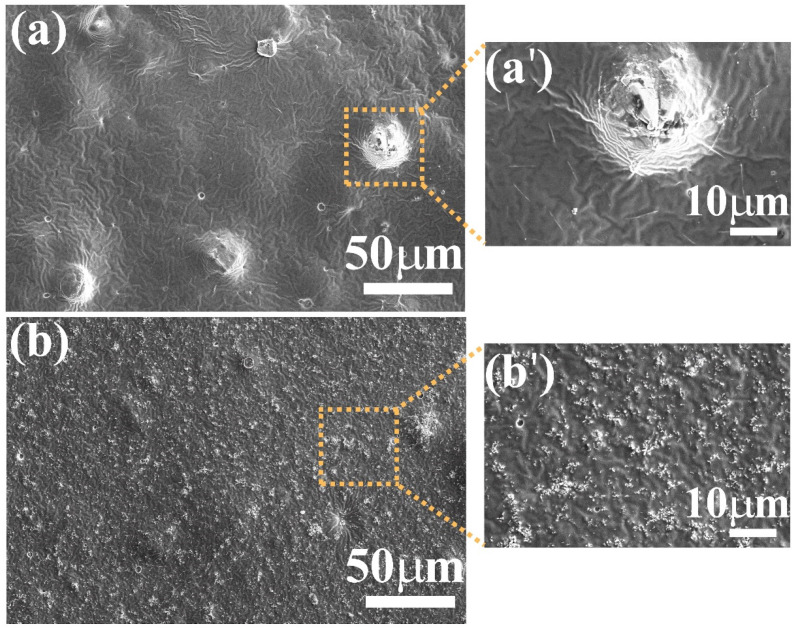
Characterization of the surface microstructure of ZnO coatings. SEM images of binder-A loaded with: (**a**) ZnO-aloe and (**b**) ZnO-starch. Magnified images (**a′**) ZnO-aloe and (**b′**) ZnO-starch were recorded in the area delimitated by the yellow dotted square in images (**a**,**b**), respectively.

The acrylic-based binder coating (binder-A) used to disperse the synthesized ZnO particles exhibits non-ordered wrinkles on the cured surface (Figure 4a); it has been reported that wrinkles appear during the drying process of double coatings when the elapsed time between the first and second coatings is too short [50]. The distribution of ZnO particles (ZnO-aloe and ZnO-starch) in binder-A was estimated from SEM images captured with SE and BSE detectors (Figure 5 and Figure S1). This showed a homogeneous dispersion of the particles without agglomerates for the surface loaded with ZnO-starch (Figure 3b and Figure S3b).

As shown in Figure 5, the surface morphology of the cured coatings containing ZnO particles depends on the synthesis route used to obtain the particles. The ZnO particles agglomerate on the coating loaded with ZnO-aloe during the curing process, forming a few tenths of micrometer clumps on the coating’s surface (Figure 5a), suggesting that the ZnO particles obtained using Aloe vera extract are not well-dispersed in binder-A due to poor interaction between the pigment and the acrylic binder, as no dispersing additives are used. These additives prevent flocculates from forming during dispersion, preventing agglomeration during the film-forming process [51]. Therefore, the examination of coating surfaces was undertaken with the BSE detector system to analyze the composition of the sample surface (Figure S1). BSE-SEM images with bright contrast reveal the presence of ZnO particles (bright white) on both surfaces (ZnO-aloe/binder-A and ZnO-starch/binder-A). However, the dispersion of ZnO-aloe particles in binder-A is much less homogeneous than the ZnO-starch dispersion.

Compared to the simple chemical composition of binder-A, the commercial white paint, paint-W, is composed of different components, such as solvents, pigments, binders, fillers, plasticizers, and additives, which control critical properties of the paint, i.e., durability, brushability, and drying time [52]. Different functional paints, such as protective, decorative, and signal-generation paints, are formulated and mixed from these common ingredients in paint [49]. These factors affect the distribution of added ZnO particles in the paint. The SEM images for the non-loaded paint-W are shown in Figure S2, where TiO_2_ particles, the majority component as a white pigment of the commercial white paint used in this work, are well distributed over the surface, with particles size ranging from 140 to 460 nm. SEM-SE and BSE mode images indicate the composition and distribution of ZnO particles in the paint-W (Figure S3). The ZnO particles in bright contrast in BSE-SEM images show a better distribution for ZnO particles obtained using starch as a fuel (Figure S3b′); the same result was observed for the coatings using binder-A, where the ZnO particles are well-dispersed with the other components present in paint-W, indicating that the ZnO-starch particles have a good interaction with the polymer matrices for coating formulation.

**Figure 6:**
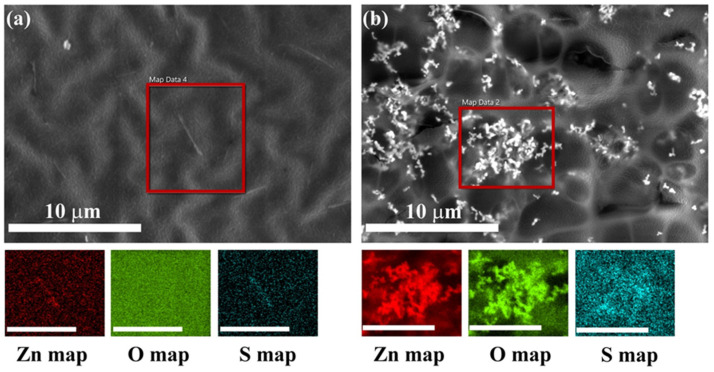
BSE-SEM image of binder-A loaded with (**a**) ZnO-aloe and (**b**) ZnO-starch particles with EDXS elemental maps of Zn, O, and S. Scale bar on the elemental maps is 5 μm. The red frame in the BSE-SEM images indicates the area of analysis.

Electron dispersive X-ray spectra confirmed the particle distribution in binder-A and paint-W observed by SEM images (Figure 6 and Figure S4). They indicate the presence of ZnO particles in the binder-A (S, Zn, and O) and the paint-W (Ti, Mg, Si, Zn, and O). The elements (Ti, Mg, and Si) are present in the paint formulation (paint-W) (Figure S2c), while the S is present in the binder-A composition (Figure 4c).

**Figure 7:**
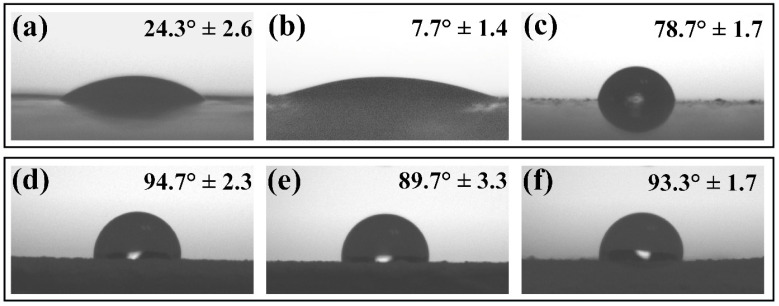
Water droplet contact angle on ZnO coatings on polycarbonate substrate: (**a**) binder-A non-loaded ZnO particles; (**b**) ZnO-aloe loaded in binder-A; (**c**) ZnO-starch loaded in binder-A; (**d**) paint-W non-loaded ZnO particles; (**e**) ZnO-aloe loaded in paint-W; and (**f**) ZnO-starch loaded in paint-W.

The virus inactivation is reported to occur when the contact of the virus is in a droplet suspension with a distinct surface. The virus inactivation performance of the surface is, therefore, highly dependent upon wetting and imbibition. Due to the presence of hydroxyl groups on the surface of ZnO, particles are intrinsically hydrophilic [53]. Thus, adding ZnO in acrylic-based paint can change the surface wettability properties of the coating, modifying the contact area between the surface and the droplet. The contact angle (CA) measurements were done on coatings loaded and non-loaded with ZnO particles. The water droplet CA on the non-loaded binder-A surface was 24.3° ± 2.6°, as shown in Figure 7a. As expected, the ZnO loaded on binder-A affected its wetting behavior. According to Figure 7b,c, the average contact angle of the water droplet on the surface loaded with ZnO-aloe and ZnO-starch in binder-A was 7.7° ± 1.4° and 78.7° ± 1.7°, respectively, indicating that the addition of ZnO-aloe particles renders the surface superhydrophilic. However, the ZnO-starch/binder-A surface tends to be hydrophobic compared to the pure binder-A surface (Figure 7a). For the surfaces coated with ZnO particles in paint-W (Figure 7d–f), the addition of ZnO-aloe particles made the surface less hydrophobic than the commercial paint-W (94.7° ± 2.3°), showing an average contact angle of 89.7° ± 3.3°. Moreover, adding ZnO-starch particles caused a minor change in the surface wettability of pure paint-W, with a contact angle of 93.3° ± 1.7°. Based on the microscopy results, it can be suggested that ZnO-aloe particles enhance the roughness of the surface compared to ZnO-starch particles since the ZnO-aloe particles are present on the very near surface region (Figure S1, see Supplementary Materials). Therefore, the particles near the surface can increase wettability and facilitate droplet spreading, increasing evaporation rate, reducing virus travel distance to the surface, and speeding up the virus interaction with the surface [54].

### 3.3. ZnO Coating Reduces Infectivity of SARS-CoV-2

**Figure 8:**
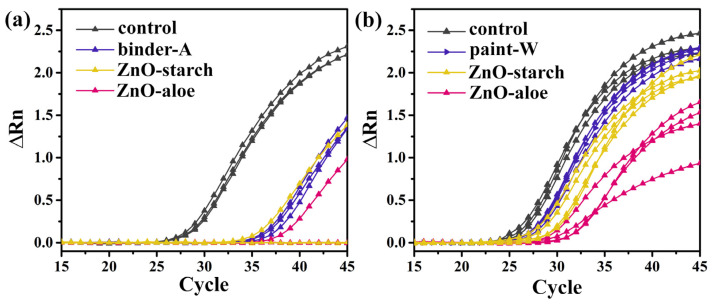
Amplification plots showing normalized reporter values (ΔRn, linear scale) as a function of the qPCR cycle for the surfaces loaded with ZnO-aloe and ZnO-starch particles using (**a**) acrylic binder (binder-A) and (**b**) white paint (paint-W) after seven days of the curing process. The amplification plot displays the product accumulation over the real-time PCR experiment based on the fluorescent signal from each sample and the cycle number.

The detection of SARS-CoV-2 RNA on surface samples indicates that the virus (viable or nonviable) was previously present on that surface. In RT-PCR assays, lower cycle thresholds (CTs) indicate higher target RNA copy numbers. To further verify the direct inhibitory effect of ZnO particles on SARS-CoV-2 of the surfaces, virus replication was detected using RT-qPCR. The virucidal exposure phase using ZnO coatings was evaluated according to the adapted ISO 22196:2011 [31]. The viral titer was significantly reduced in SARS-CoV-2 infected cells treated for 24 h with ZnO-aloe and ZnO-starch coated in two waterborne acrylic paints (binder-A; and paint-W), compared with the level in infected cells on non-loaded coating (Figure 5, inset the control in black color). Our results show that SARS-CoV-2 infectivity was inactivated at 99.8% and 98.7% by 24 h of exposure to the ZnO-aloe and ZnO-starch, respectively when the ZnO particles were coated in binder-A (Figure 8a). When coated in paint-W, the infectivity values decreased, showing a reduction in the viral load of the surfaces after the exposure of 94.6% and 67.3% to the ZnO-aloe and ZnO-starch, respectively (Figure 8b). The results indicate that commercial white paint (paint-W), even with TiO_2_ in its formulation, has low antiviral activity against the SARS-CoV-2 virus but increased activity with ZnO synthesized in this work. The virus infectivity of a surface has been reported to be related to the surface wettability of the samples [55,56]. In this context, the cured ZnO-aloe in binder-A shows a lower contact angle; in other words, higher wettability for effective contact promoting immediate and intimate contact with viral contaminants. The high Ct values shown in Table 1 for the pure binder-A may be associated with tests conducted on incompletely cured surfaces, as no additives are present in the chemical composition of binders. Their curing process is longer than paints; therefore, their surface chemistry may interact with the medium virus solution. The antiviral test was performed for the best surface result, ZnO-aloe/binder-A, to verify these results after one month of curing (Figure S5). Upon exposure to the surface containing ZnO-aloe particles, the percentage of viral load reduction after 24 h was 80.9%. At the same time, pure binder-A showed a low viral load reduction (Table S1), thus denoting the efficiency of ZnO-aloe as an antiviral pigment.

The number of copies of the genes of SARS-CoV-2 in the ZnO-treated infected cells decreased significantly compared with the control sample levels, indicating that ZnO-aloe/binder-A significantly inhibited the better replication of SARS-CoV-2 in the studied coated surfaces. Furthermore, it was found that the virus titer decreased further with the application of ZnO particles in binder-A.

## 4. Conclusions

The reported synthesis routes to obtain ZnO particles and their dispersion in paint are low-cost and suitable for large-scale manufacturing. An acrylic binder and a commercial white paint were used to obtain the ZnO-based coatings to evaluate their efficiency against SARS-CoV-2. Morphological characterization shows particles uniformly distributed on the sample surface coated with ZnO-starch particles in binder-A and paint-W, indicating that ZnO particles obtained using starch interact well with acrylic-based coatings, independent of additives. On the contrary, for both coatings, ZnO-aloe exhibited massive aggregation.

The ZnO particles dispersed in both coatings display virucidal activity according to RT-PCR results evaluated within 24 h, with the effective result coming from ZnO-aloe added to binder-A (prod. Amsterdam). The results also showed that the RT-PCR method could be used to determine the presence of SARS-CoV-2 on surfaces. Moreover, ZnO particles have the potential application as a pigment in acrylic-based coatings, which may reduce infection and limit the spread of SARS-CoV-2 via the fomite route. Because acrylic-based coatings are widely used outdoors and indoors, these coatings with added ZnO particles are promising materials to be applied in civil constructions.

## Figures and Tables

**Table 1:** Decrease of viral load of samples and controls after 24 h of exposure determined by RT-qPCR.

Sample	% Reduction
Copper (Control)	99.9
Binder-A	85.6
ZnO-aloe (binder-A)	99.8
ZnO-starch (binder-A)	98.7
Paint-W	44.9
ZnO-aloe (paint-W)	94.6
ZnO-starch (paint-W)	67.3

Note: The exposure phase of the tests was done in a BSL2 lab under conditions identical to COVID-19 screening conditions. No virus amplification or experimental steps other than those for COVID-19 screening was performed.

## Data Availability

The data presented in this study are available on request from the corresponding author.

## Supplementary Materials

The following supporting information can be downloaded at: https://www.mdpi.com/article/10.3390/nano12234345/s1, Figure S1: SEM images of ZnO particles dispersed in binder-A captured with a backscattered electron (BSE) detector at low magnification: (a) ZnO-aloe; and (b) ZnO-starch. Figure S2: SEM images of commercial white paint surface (paint-W) (a) captured with a secondary electron detector (SE), (b) captured with a backscattered electron (BSE) detector, and (c) SEM image of paint-W with EDXS elemental maps of C, Ti, Si, and O. Scale bar on the elemental maps is 25 μm. The red frame in the SEM image indicates the area of analysis; Figure S3: SEM images of coating surfaces of paint-W loaded with ZnO-aloe and ZnO-starch particles: (a) (ZnO-aloe) and (b) (ZnO-starch) were captured with a secondary electron detector. In contrast, images (a′) (ZnO-aloe) and (b′) (ZnO-starch) were captured with a back-scattered electron detector; Figure S4: SEM image of paint-W loaded with (a) ZnO-aloe and (b) ZnO-starch with EDXS elemental maps of Zn, O, Ti and Si. The scale bar on the elemental maps is 25 μm. The red frames in the SEM images indicate the area of analysis; Figure S5: Displays amplification plots showing normalized reporter values (ΔRn, linear scale) as a function of the qPCR cycle for the surfaces loaded with ZnO-aloe particles after 30 days of the curing process. The amplification plot displays the product accumulation over the real-time PCR experiment based on the fluorescent signal from each sample and the cycle number; Table S1: Decrease of viral load of samples and controls after 24 hours of exposure determined by RT-qPCR.
