# Supplementary material for: Antiviral Properties against SARS-CoV-2 of Nanostructured ZnO Obtained by Green Combustion Synthesis and Coated in Waterborne Acrylic Coatings
Source: Nanomaterials (Basel). 2022 Dec 6;12(23):4345. doi: 10.3390/nano12234345 (PMC9740257; doi:10.3390/nano12234345)
Supplement: Supplementary file 1 [file nanomaterials-12-04345-s001.zip › nanomaterials-2032229-supplementary.pdf]

nanomaterials

MDPI

Supplementary Materials

Antiviral Properties against SARS-CoV-2 of Nanostructured ZnO Obtained by Green Combustion Synthesis and Coated in Waterborne Acrylic Coatings

Julia de O. Primo [1,2], Jamille de S. Correa [1], Dienifer F. L. Horsth [1,2], Arkaprava Das [2], Marcin Zając [3], Polona Umek [4], Ruddy Wattiez [5], Fauze J. Anaissi [1], Rob C. A. Onderwater [6] and Carla Bittencourt [2,*]

[1] Departamento de Química, Universidade Estadual Do Centro-Oeste, 85-040-200 Guarapuava, Brazil
[2] Chimie des Interactions Plasma-Surface (ChIPS), Research Institute for Materials Science and Engineering, University of Mons, 7000 Mons, Belgium
[3] National Synchrotron Radiation Centre Solaris, Jagiellonian University, 30-392 Kraków, Poland
[4] Solid State Physics Department, Jožef Stefan Institute, 1000 Ljubljana, Slovenia
[5] Department of Proteomics and Microbiology, University of Mons, 7000 Mons, Belgium
[6] Materia Nova ASBL, 7000 Mons, Belgium
* Correspondence: carla.bittencourt@umons.ac.be

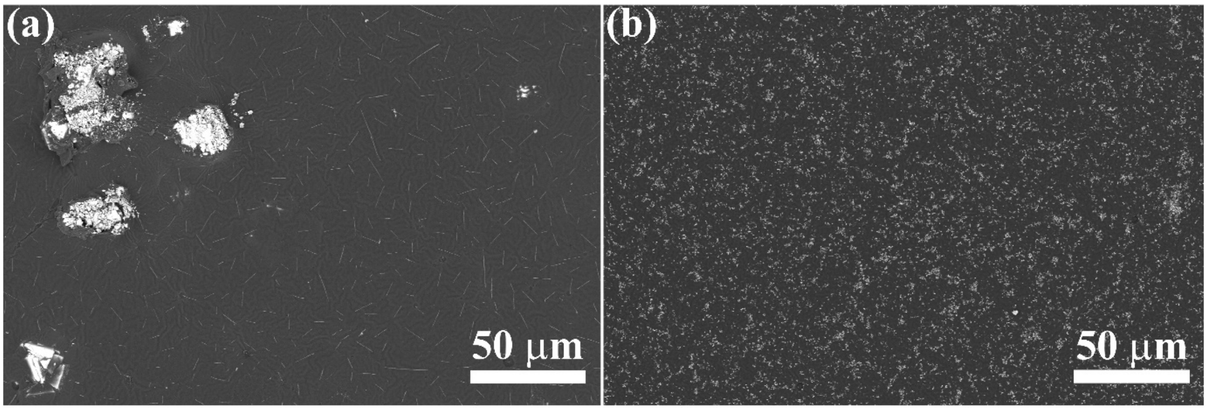

Figure S1. SEM images of ZnO particles dispersed in binder-A captured with a backscattered electron (BSE) detector at low magnification: (**a**) ZnO-aloe; and (**b**) ZnO-starch.

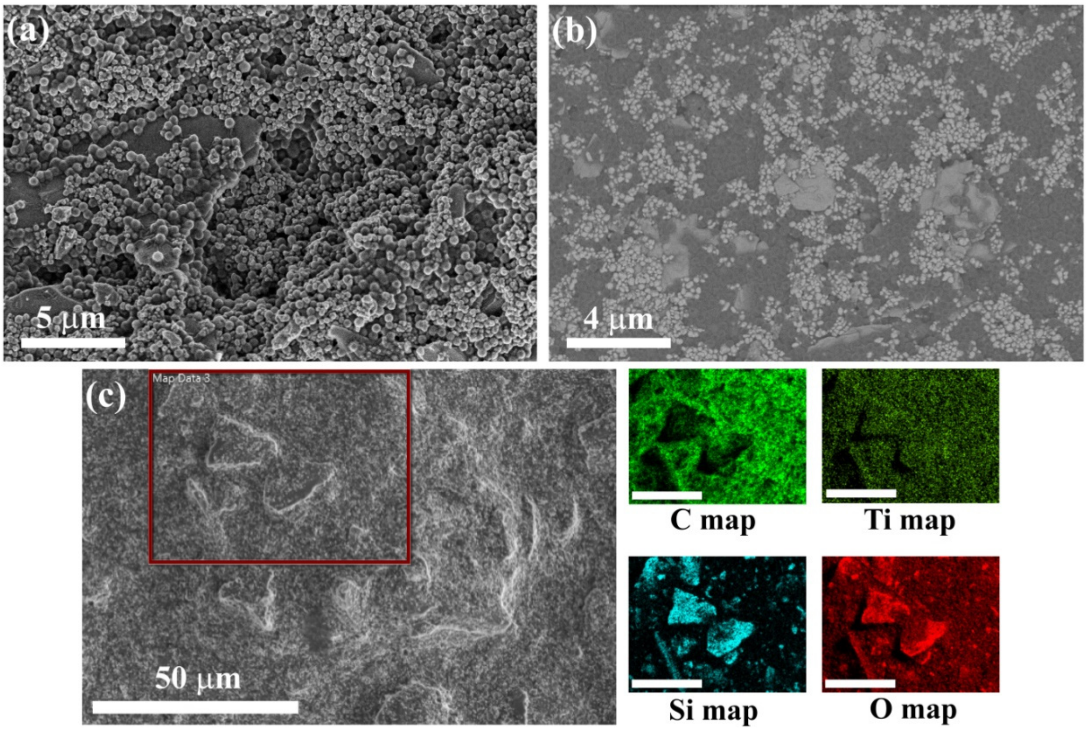

Figure S2. SEM images of commercial white paint surface (paint-W) (**a**) captured with a secondary electron detector (SE), (**b**) captured with a backscattered electron (BSE) detector, and (**c**) SEM image of paint-W with EDXS elemental maps of C, Ti, Si, and O. Scale bar on the elemental maps is 25 µm. The red frame in the SEM image indicates the area of analysis.

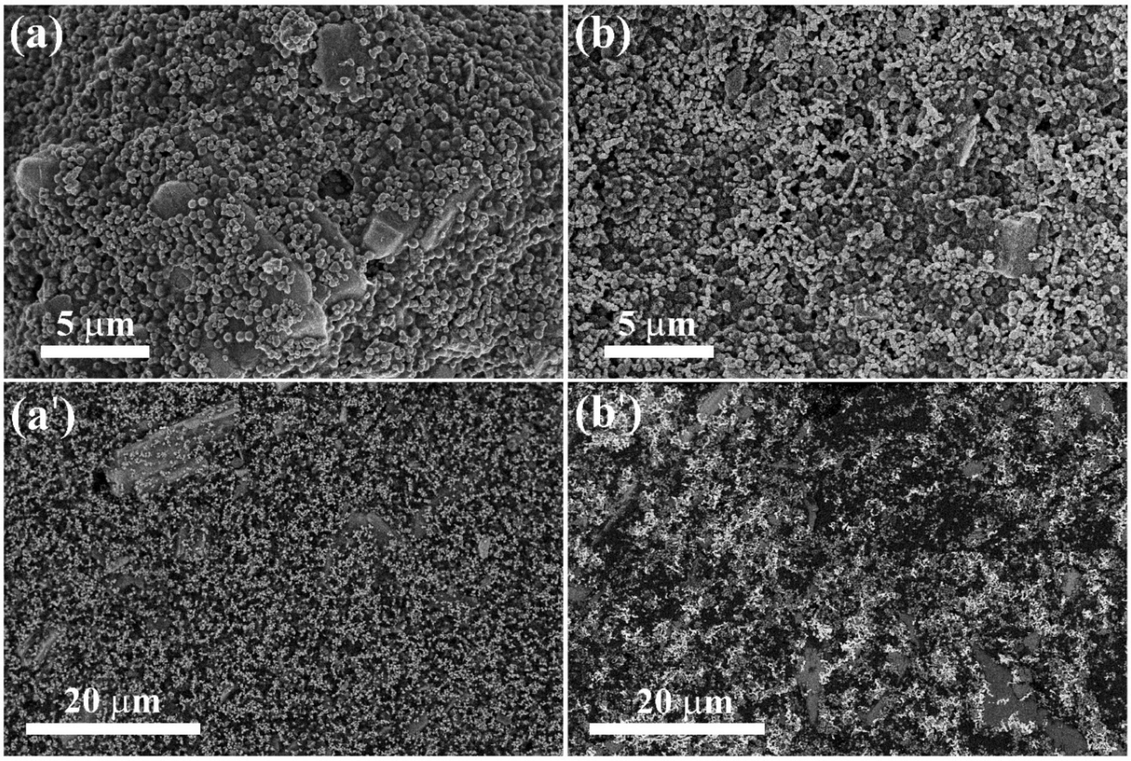

Figure S3. SEM images of coating surfaces of paint-W loaded with ZnO-aloe and ZnO-starch particles: (**a**) (ZnO-aloe) and (**b**) (ZnO-starch) were captured with a secondary electron detector. In contrast, images (**a′**) (ZnO-aloe) and (**b′**) (ZnO-starch) were captured with a back-scattered electron detector.

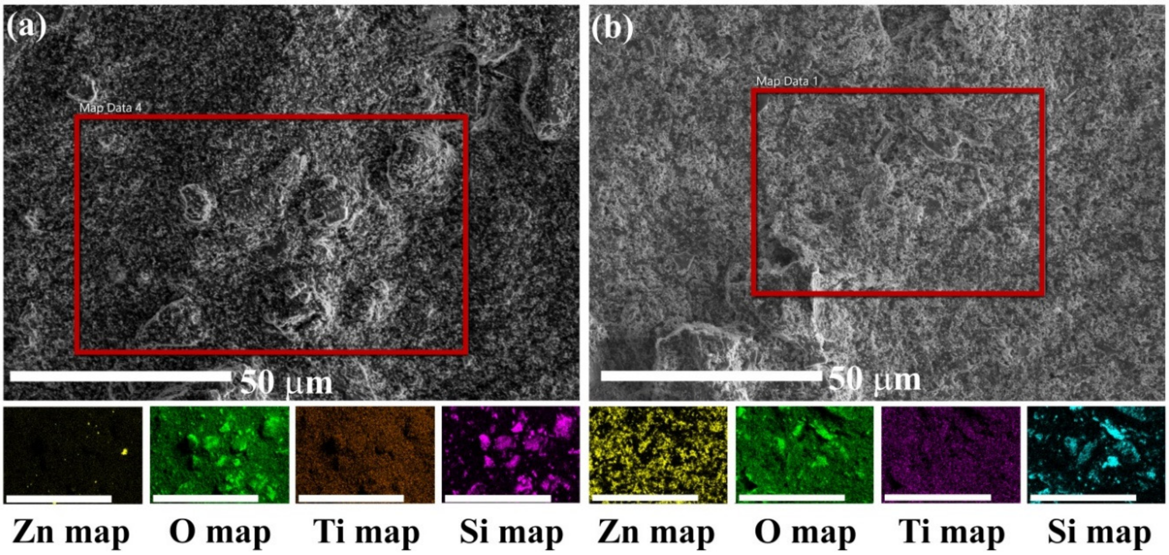

Figure S4. SEM image of paint-W loaded with (**a**) ZnO-aloe and (**b**) ZnO-starch with EDXS elemental maps of Zn, O, Ti and Si. The scale bar on the elemental maps is 25 μm. The red frames in the SEM images indicate the area of analysis.

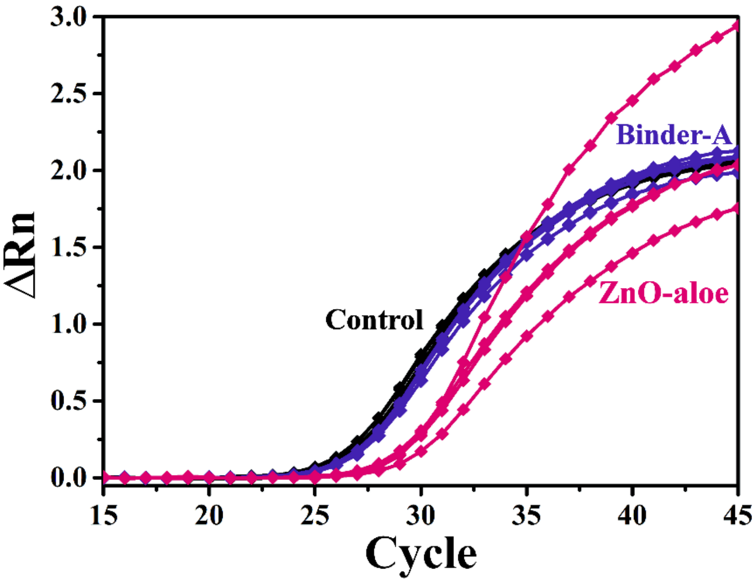

Figure S5. Displays amplification plots showing normalized reporter values (ΔRn, linear scale) as a function of the qPCR cycle for the surfaces loaded with ZnO-aloe particles after 30 days of the curing process. The amplification plot displays the product accumulation over the real-time PCR experiment based on the fluorescent signal from each sample and the cycle number.

Table S1. Decrease of viral load of samples and controls after 24 hours of exposure determined by RT-qPCR.

Sample	% Reduction
Copper (Control)	99.9
Binder-A	18.0
ZnO-AV (binder-A)	81.5

Note: The exposure phase of the tests was done in a BSL2 lab under conditions identical to COVID screening conditions. No virus amplification or experimental steps other than those for COVID screening was performed.
